# Performance evaluations of ChatGPT models for clinical applications in psychiatry using the OpenAI HealthBench Framework: Evidence of domain-specific limitations

**DOI:** 10.1192/j.eurpsy.2026.11515

**Published:** 2026-07-31

**Authors:** A. Smith, J. Grana, S. Hachen, E. Schollerer, A. Buadze, M. Liebrenz

**Affiliations:** 1 Department of Forensic Psychiatry, University of Bern, Bern; 2 Psychiatric Hospital, Zurich, University of Zurich, Zurich, Switzerland

## Abstract

**Introduction:**

Although Large Language Model (LLM) deployment in healthcare continues to advance, systematic measurements of their performance for clinical contexts and use-cases in psychiatry remains limited. OpenAI’s HealthBench framework comprises standardised evaluation metrics for its utility and efficacy in healthcare, yet whether these general benchmarks adequately assess psychiatric competencies remains empirically untested.

**Objectives:**

This study sought to evaluate ChatGPT iterations (GPT-3.5 through to GPT-o3) on psychiatry-relevant conversations extracted from the HealthBench dataset, quantifying performance trajectories and identifying domain-specific limitations.

**Methods:**

147 psychiatry-related dialogues with LLMs from 5,000 HealthBench conversations were systematically extracted using keyword taxonomy and a manual human review. Based off this, three evaluation datasets were analysed: HealthBench-Psychiatry (n=147), HealthBench-Psychiatry Consensus (n=104), HealthBench-Psychiatry Hard (n=38). A rubric-based assessment was conducted for five behavioral axes and seven psychiatrically-relevant themes using OpenAI’s evaluation framework.

**Results:**

Overall, evaluative scores for psychiatric conversations scores increased between models from 0.15 (GPT-3.5) to 0.59 (GPT-o3), in line with general medical benchmarks for LLM performance (mean deficit: -0.03). However, importantly, the pre-defined HealthBench domains for “instruction following” and “context awareness” demonstrated a lack of statistically significant improvements across recent iterations (p>0.344).

**Conclusions:**

This study demonstrates marked improvements in ChatGPT psychiatric performance between iterations, aligning with broader medical benchmarks. Yet, persistent plateaus highlight critical gaps in meeting the nuanced demands of psychiatric clinical tasks. Accordingly, these findings suggest that current LLMs remain limited in their suitability for direct clinical deployment in mental health settings. Future progress will require domain-specific fine-tuning, rigorous psychiatric benchmarking, and safeguards to ensure safe, contextually sensitive integration into psychiatric practice.

**Disclosure of Interest:**

None Declared

